# Solubility and Stability Enhanced Oral Formulations for the Anti-Infective Corallopyronin A

**DOI:** 10.3390/pharmaceutics12111105

**Published:** 2020-11-18

**Authors:** Anna K. Krome, Tim Becker, Stefan Kehraus, Andrea Schiefer, Christian Steinebach, Tilman Aden, Stefan J. Frohberger, Álvaro López Mármol, Dnyaneshwar Kapote, Rolf Jansen, Lillibeth Chaverra-Muñoz, Marc P. Hübner, Kenneth Pfarr, Thomas Hesterkamp, Marc Stadler, Michael Gütschow, Gabriele M. König, Achim Hoerauf, Karl G. Wagner

**Affiliations:** 1 Department of Pharmaceutical Technology and Biopharmaceutics, University of Bonn, 53121 Bonn, Germany; krome@uni-bonn.de (A.K.K.); tim.becker@uni-bonn.de (T.B.); alvaro.lopez@uni-bonn.de (Á.L.M.); dkapote@uni-bonn.de (D.K.); 2 Institute for Medical Microbiology, Immunology and Parasitology, University Hospital Bonn, 53127 Bonn, Germany; andrea.schiefer@uni-bonn.de (A.S.); tilman.aden@ukbonn.de (T.A.); stefan.frohberger@gmx.de (S.J.F.); huebner@uni-bonn.de (M.P.H.); kenneth.pfarr@ukbonn.de (K.P.); hoerauf@uni-bonn.de (A.H.); 3 German Center for Infection Research (DZIF), Partner Site Bonn-Cologne, 53127 Bonn, Germany; skehraus@uni-bonn.de; 4 Institute for Pharmaceutical Biology, University of Bonn, 53115 Bonn, Germany; g.koenig@uni-bonn.de; 5 Pharmaceutical & Medicinal Chemistry, University of Bonn, 53121 Bonn, Germany; c.steinebach@uni-bonn.de (C.S.); guetschow@uni-bonn.de (M.G.); 6 Department of Microbial Drugs, Helmholtz Centre for Infection Research, 38124 Braunschweig, Germany; rolf.jansen@helmholtz-hzi.de (R.J.); lillibeth.chaverra-munoz@helmholtz-hzi.de (L.C.-M.); Marc.Stadler@helmholtz-hzi.de (M.S.); 7 German Center for Infection Research (DZIF), Partner Site Hannover-Braunschweig, 38124 Braunschweig, Germany; 8 Translational Project Management Office (TPMO), German Center for Infection Research (DZIF), 38124 Braunschweig, Germany; Thomas.Hesterkamp@helmholtz-hzi.de

**Keywords:** corallopyronin A (CorA), antibiotic, anthelmintic, povidone (PVP), copovidone (PVP/VA), solubility enhanced formulation, stability enhanced formulation, spray-dried amorphous solid dispersion (ASD), biphasic dissolution (BiPHa+), pharmacokinetic analysis

## Abstract

Novel-antibiotics are urgently needed to combat an increase in morbidity and mortality due to resistant bacteria. The preclinical candidate corallopyronin A (CorA) is a potent antibiotic against Gram-positive and some Gram-negative pathogens for which a solid oral formulation was needed for further preclinical testing of the active pharmaceutical ingredient (API). The neat API CorA is poorly water-soluble and instable at room temperature, both crucial characteristics to be addressed and overcome for use as an oral antibiotic. Therefore, amorphous solid dispersion (ASD) was chosen as formulation principle. The formulations were prepared by spray-drying, comprising the water-soluble polymers povidone and copovidone. Stability (high-performance liquid chromatography, Fourier-transform-infrared spectroscopy, differential scanning calorimetry), dissolution (biphasic dissolution), and solubility (biphasic dissolution, Pion’s T3 apparatus) properties were analyzed. Pharmacokinetic evaluations after intravenous and oral administration were conducted in BALB/c mice. The results demonstrated that the ASD formulation principle is a suitable stability- and solubility-enhancing oral formulation strategy for the API CorA to be used in preclinical and clinical trials and as a potential market product.

## 1. Introduction

In addition to better prevention of infectious diseases and appropriate use of existing antibacterial drugs, novel antibiotics are urgently needed to combat the increase in morbidity and mortality due to resistant bacteria [1,2,3,4]. The World Health Organization published information about the current clinical and preclinical antibiotic pipeline: In 2020 only 50 antibiotics were in the clinical antibiotic pipeline globally, an insufficient number to tackle the observed rise of infections due to antimicrobial resistance [5,6]. Therefore, more research towards new antibiotics and further development of the antibiotics in the preclinical pipeline is vital. One of the antibiotics currently in the late preclinical stage is corallopyronin A (CorA).

**Figure 1:**
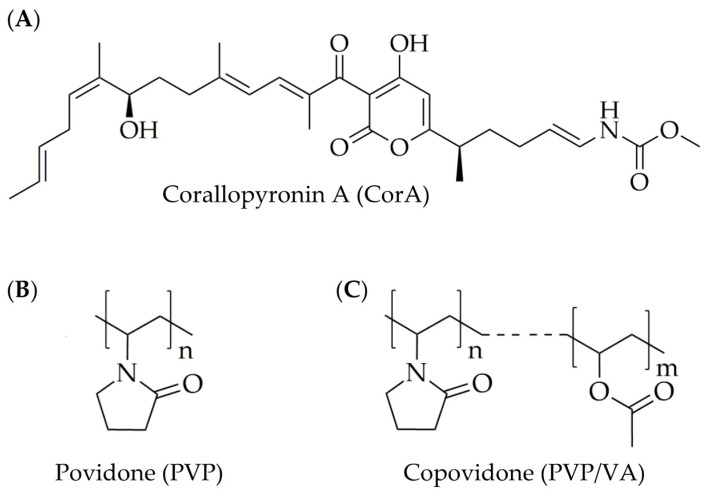
Chemical structures of the natural antibiotic corallopyronin A (CorA) (**A**), and the polymers povidone (PVP) (**B**) and copovidone (PVP/VA) (**C**).

CorA (Figure 1A) is a natural product derived from cultures of the Myxobacterium *Corallococcus coralloides*. The active pharmaceutical ingredient (API) was found in 1985 in the course of a screening program for antibiotics from Myxobacteria at the Helmholtz Centre for Infection Research (the former biotechnological research facility Gesellschaft für Biotechnologische Forschung, Braunschweig, Germany). CorA was shown to inhibit the bacterial DNA-dependent RNA polymerase. It has antibacterial activity against Gram-positive and some Gram-negative pathogens, e.g., *Chlamydia trachomatis*, *Orientia tsutsugamushi*, *Staphylococcus aureus*, and *Wolbachia*. Based on its highly effective in vivo depletion of *Wolbachia* endobacteria from filarial nematodes, the antibiotic was selected as a preclinical trial candidate with the aim to develop it to treat human filarial infections [7,8,9,10,11,12,13,14,15,16].

Prior unpublished material characterization experiments showed that the neat API CorA exhibited sufficient permeability but poor water solubility. Additionally, instability and pronounced isomerization occurred when stored at room temperature. The API CorA was amorphous in a semisolid state, adhesive and of a waxy consistency, which was not feasible for direct and easy processing into solid dosage form for oral administration. Efforts to crystalize the drug have failed. No solid pharmaceutical formulations of CorA were available. Bioavailability seemed to be limited by solubility rather than absorption.

The objective of the present study was to provide a solid oral formulation of CorA by employing the amorphous solid dispersion (ASD) principle simultaneously addressing the two main challenges: Improving solubility and stability. For ASD formulations, solubility enhancement has been described by many authors for other APIs [17,18,19,20], as has stability enhancement [21,22,23]. Therefore, we hypothesized that CorA-ASDs could provide a mechanism to enhance both solubility and stability. A stabilization mechanism for CorA, due to reduced intermolecular mobility of CorA in the polymer matrix, possibly stabilizing molecular interactions between CorA and the polymer, thus diminishing isomerization. Since CorA was adhesive and of a waxy consistency, sample preparation via prior dissolution in an organic solvent and subsequent spray-drying was chosen to formulate the ASDs. The aqueous soluble polymers povidone (PVP) and copovidone (PVP/VA) were selected (Figure 1B,C). Analysis of the formulations evaluating the stability, dissolution and solubility properties were performed by high-performance liquid chromatography (HPLC), differential scanning calorimetry (DSC), Fourier-transform-infrared spectroscopy (FT-IR) and by an in vivo predictive biphasic dissolution BiPHa+ [24]. Pharmacokinetic evaluations were conducted in BALB/c mice.

## 2. Materials and Methods

### 2.1. Materials

CorA Production: For the media casein peptone and agar were purchased from BD Biosciences GmbH (Heidelberg, Germany). Tris(hydroxymethyl)aminomethane (TRIS), potassium dihydrogen phosphate, calcium chloride dihydrate, potassium acetate, 2-(4-(2-hydroxyethyl)-1-piperazinethanesulfonic acid (HEPES) and vitamin B12 were purchased from Carl Roth GmbH & Co. KG (Karlsruhe, Germany). Magnesium sulfate heptahydrate was purchased from VWR Chemicals (Langenfeld, Germany). Defatted soy flour and glucose monohydrate were purchased from Cargill Deutschland GmbH (Krefeld, Germany). Yeast extract was purchased from Ohly GmbH (Hamburg, Germany). Iron chloride, 1-docosanol and kanamycin were purchased from Merck KGaA (Darmstadt, Germany). Vanillic acid and Amberlite XAD-16 resin were purchased from Sigma-Aldrich Chemie GmbH (Steinheim, Germany).

Physicochemical Characterization: Potassium chloride (analytical grade), 0.5 M hydrochloric acid, sodium hydroxide concentrate and 1-octanol were purchased from Sigma-Aldrich Chemie GmbH.

CorA Formulations: Polyvinylpyrrolidone (Kollidon^®^ 30 LP) was kindly provided by BASF (Ludwigshafen, Germany), vinylpyrrolidone-vinyl acetate copolymer (VIVAPHARM^®^ PVP/VA 64) was kindly provided by JRS PHARMA (Rosenberg, Germany). Ethanol 99.8% was purchased from Carl Roth, propylene glycol from Caesar & Lorenz GmbH (Hilden, Germany), and Kolliphor^®^ HS-15 was from Sigma-Aldrich Chemie GmbH.

^1^H NMR and HPLC Quantification: Acetonitrile-*d*_3_ (99.8 atom% D) and D_2_O (99.9 atom% D) were purchased from Deutero GmbH (Kastellaun, Germany), acetonitrile LC-MS grade and water LC-MS grade were purchased from Bernd Kraft GmbH (Duisburg, Germany), and ammonium acetate from Merck KGaA.

Biphasic Dissolution Test: Tri-potassium phosphate, lecithin, 1-decanol, and sodium taurocholate were purchased from Alfa Aesar GmbH & Co. KG (Kandel, Germany), acetic acid from Chemie GmbH (Steinheim, Germany), tri-potassium citrate was from Carl Roth, and sodium hydroxide was purchased from VWR Chemicals (Darmstadt, Germany).

### 2.2. Production of CorA

CorA was produced by fermentation of the natural producer strain *Corallococcus coralloides* B035 or from the heterologous host *Myxococcus xanthus* strain DK1622 pDPO mxn16 Tpase [25]. The natural producer as well as the heterologous host were maintained as cryo-cultures at −80 °C before they were used to inoculate agar plates of CTT medium [25] supplemented with kanamycin. The plates were incubated at 30 °C for 72 h. Cells from the surface of grown plates were used to inoculate the first liquid seed culture consisting of M7/S4 medium [25]. The culture was incubated at 30 °C and 180 revolutions per minute (rpm) in an orbital shaker (Multitron Pro, Infors HT AG, Basel, Switzerland) for 48 h. The second seed culture M7/S4 medium [25] was inoculated with the first seed culture and incubated at the same cultivation conditions for another 48 h. The transfer to the seed bioreactor was done by inoculating the second seed culture into M7/S4 medium [25] without HEPES in a 15 L stirred tank bioreactor (C10-3, BBI-Biontech GmbH, Berlin, Germany). Set point for temperature was 30 °C, for pH 7.4 ± 0.1, and aeration of 0.005 volume of air per volume of liquid per minute (vvm). The pO_2_ was maintained at 20% by increasing the stirring speed using three Rushton impellers. The production process was performed with M7/S6 medium [25] supplemented with vanillic acid in a 150 L stirred tank bioreactor (9-4233, Chemap AG, Volketswil, Switzerland). Process parameters were maintained at the same levels as in the seed bioreactors and the cultivation was carried out for 144 h until the harvest.

The supernatant of the fermentation was stirred with Amberlite XAD-16 resin for the extraction of CorA. The resin was recovered by sieving and purged with buffered 50% aqueous methanol, before enriched CorA was eluted with buffered methanol. Evaporation of methanol, and extraction of the products from the diluted remaining buffer with ethyl acetate provided raw CorA. Isolation of pure CorA (90–99%) was achieved by reversed phase chromatography on a medium pressure liquid chromatography (MPLC) column YMC OSD-AQ (12 nm, 20 µm) (YMC Europe GmbH, Dinslaken, Germany) with 30% buffered aqueous acetonitrile.

### 2.3. Content Assay of CorA by ^1^H NMR

The content measurement of CorA was determined by ^1^H NMR spectrometry at 300 MHz using a Bruker Avance DPX300 NMR spectrometer. CorA (10–30 mg) and the internal reference dimethyl sulfone (2–4 mg) were weighed accurately to 0.1 mg and dissolved in acetonitrile-*d*_3_ (600 µL) and D_2_O (100 µL). For the measurement, 128 scans at a relaxation time of 30 s and a line broadening factor of 0.3 Hz were conducted. Baseline and phase correction were performed and the singlet of the reference compound and the analyte singlet of H-5 of CorA were quantified. The content of the analyte was calculated according to Equation (1).

(1) $$C_{\mathrm{corA}}\left(\%\right)=C_{\mathrm{Ref}.}\left(\%\right)\times \frac{I_{\mathrm{CorA}}\times {\mathrm{No}}_{\mathrm{Ref}.}\times {\mathrm{MW}}_{\mathrm{CorA}}\times m_{\mathrm{Ref}.}}{I_{\mathrm{Ref}.}\times {\mathrm{No}}_{\mathrm{CorA}}\times {\mathrm{MW}}_{\mathrm{Ref}.}\times m_{\mathrm{CorA}}}$$

Equation (1). Content quantification for CorA-batches. C = content, CorA = corallopyronin A, Ref. = internal reference (dimethyl sulfone), I = signal intensity, No = number of protons (dimethyl sulfone; *δ* 2.99, 6H; CorA: *δ* 6.06, 1H, MW = molecular weight (dimethyl sulfone: 94.13 g/mol; CorA: 527.65 g/mol), m = sample weight) 

### 2.4. Physicochemical Characterization of CorA via Pion’s T3 Apparatus

Determinations of the negative log of the acid dissociation constant (p*K*a), the pH-dependent aqueous solubility, the partition coefficient (log *P*) and the distribution coefficient (log *D*) were performed on Pion’s SirusT3 apparatus (Pion Inc., Forest Row, UK), via potentiometric and UV-metric standard methods at 25 °C [26,27,28,29,30].

### 2.5. Preparation of the Spray-Dried CorA-ASD Formulations

CorA and the water-soluble polymer PVP or PVP/VA were dispensed, ethanol was added, and the mixture was placed into an ultrasonic bath (<25 °C) (Sonorex Digitec, Bandelin electronic GmbH & Co. KG, Berlin, Germany) until CorA and the polymer were fully dissolved. A solubility test of CorA in ethanol demonstrated a solubility of >100 mg/mL at 20 °C. Spray-drying was performed using a B-290 mini spray dryer (BÜCHI, Essen, Germany) with the aspirator set to 100% and the pump running at 20%. Nitrogen was used as inert drying gas. Further process information is listed in Table 1. Spray-drying yielded an amorphous solid powder of CorA embedded in water-soluble polymer PVP or PVP/VA.

### 2.6. Stability Analysis of CorA by HPLC-DAD

The stability of neat CorA and the ASD formulations of CorA were analyzed after storage of the samples under different stability conditions (25 °C/60% RH, 30 °C/65% RH, and 40 °C/75% RH) for one, two, four weeks and three months. Twenty-five degrees Celsius and 60% RH was maintained in a stability chamber (KBF 720, Binder GmbH, Tuttlingen, Germany). All other humidity levels were set using saturated salt solutions (sodium nitrite for 65% RH and sodium chloride for 75% RH) in closed glass desiccators, which were stored in the drying cabinets (UM 400, Memmert GmbH + Co. KG, Schwabach, Germany) for temperature control. The samples were stored in closed twist-off glass vials, and in the presence of silica desiccant, since the polymers PVP and PVP/VA are hygroscopic [31]. Neat CorA samples were stored under air or nitrogen and the CorA-ASDs were stored under nitrogen. The CorA content was analyzed by high-performance liquid chromatography with an Alliance e2695 separation module and a Diode-Array Detector (2998 PDA detector) (Waters, Eschborn, Germany) (HPLC-DAD). For the HPLC-DAD analysis, the samples were dissolved in acetonitrile. A Waters XBridge^®^ Shield RP18 column (3.5 µm, 2.1 × 100 mm, 130 A) was used at 30 °C. Mobile phases A (acetonitrile/water 5/95 with 5 mM ammonium acetate and 40 µL acetic acid per Liter) and B (acetonitrile/water 95/5 with 5 mM ammonium acetate and 40 µL acetic acid per Liter) were used in the solvent gradient from 70%A/30%B to 20%A/80%B, stepwise (Table A1) within 30 min and a flow rate of 0.3 mL/min. CorA was precisely quantified via an external reference standard measured at 300 nm.

### 2.7. DSC Analysis

The DSC measurements were performed with a Mettler Toledo DSC 2 (Gießen, Germany) and analyzed using STAR^e^ software (Version 13.00 a, Mettler Toledo, Gießen, Germany, 2014). Approximately 6–8 mg of a sample was filled in an aluminum crucible with a pierced lid. The samples were heated from –30 to 100 °C for the neat CorA sample, from 0 to 190 °C for the neat PVP sample, from 0 to 170 °C for the PVP-ASD samples, from 0 to 150 °C for the neat PVP/VA and from 0 to 140 °C for the PVP/VA-ASD samples. The multi-frequency temperature modulation (TOPEM-mode) with a heating rate of 2 K/min with a dry nitrogen-purge of 30 mL/min was used.

### 2.8. FT-IR Analysis

IR spectra were recorded using a FT-IR Spectrum BX spectrometer (Perkin Elmer, Rodgau, Germany), interfaced with a spectra golden gate diamond ATR system. Data evaluation was performed by Perkin-Elmer Spectrum software (Version 3.01, Perkin-Elmer, Rodgau, Germany, 1999).

### 2.9. Biphasic Dissolution Tests via BiPHa+

**Figure 2:**
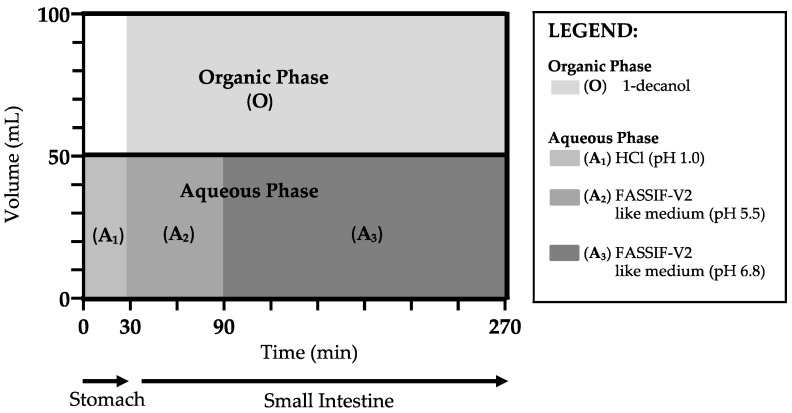
Media setup of the biphasic dissolution model BiPHa+.

The neat CorA and CorA-ASD formulations were investigated using the biphasic dissolution apparatus BiPHa+ [24] (Figure 2). For this purpose, 50 mL of HCl (0.1 M) were filled in a cylindrical vessel with a diameter of 5 cm and kept at a temperature of 37 °C for the total dissolution. The samples were prepared by weighing out 10 mg neat API or 50 mg ASD formulation. The samples were then added into the vessel. The hydrodynamic effect was achieved by triangle magnetic stirrers. After 30 min (representing the stomach passage), FaSSIF-V2 like concentrate [24] was added to the aqueous phase simultaneously to the first pH-shift from pH 1.0 to 5.5 (simulating the upper small intestine), and 50 mL of 1-decanol was added automatically above the aqueous phase. After 90 min the next pH-shift from pH 5.5 to 6.8 after 90 min was adjusted gradually (simulating the lower small intestine). Both pH-shifts were caused by adding a respective amount of McIlvaine buffer [24]. The complete dissolution took 4.5 h. The concentration profiles of both the aqueous and organic phases were measured continuously with an 8454 UV-Vis spectrophotometer (Agilent, Waldbronn, Germany) at 394 nm in the organic phase and in the aqueous phase at 325 nm (pH 1) and 336 nm (pH 5.5–6.8) and quantified via external calibration curves. Three independent dissolution tests were performed for each sample.

### 2.10. Pharmacokinetic Study-Setup and Plasma Sample Analysis

The animal experiment was conducted according to European Union Directive 2010/63/EU and was approved by the State Agency for Nature, Environment, and Consumer Protection of North Rhine-Westphalia (LANUV), Germany (AZ 84-02.04.2015.A507). Female BALB/c mice (7–10 weeks old) were obtained from Janvier (Le Genest-Saint-Isle, France). Animals were housed at the animal facility of the Institute for Medical Microbiology, Immunology and Parasitology at the University Hospital Bonn, Germany.

Pharmacokinetic analysis was performed in fed mice (*n* = 4). Due to the small anatomy of mice, the oral administration of the solid CorA-PVP-ASD formulation could only be administered as a suspension. Therefore, the solid CorA-PVP formulation was suspended in PBS (phosphate-buffered saline; pH = 7.4) immediately before the administration via gavage (CorA 36 mg/kg, volume 10 mL/kg). Blood samples from the vena facialis were collected after 5, 10, 15, 30, 180, and 480 min. The samples were centrifuged for 10 min at 4 °C at 3220× *g* (Centrifuge Fresco 17, Thermo Fisher Scientific, Vienna, Austria). The generated plasma was mixed in a ratio of 1:3 with ice-cold acetonitrile. The mixture was vortexed for 10 s (Vortex RS-VF 10, Phoenix Instrument, Garbsen, Germany) and centrifuged for 25 min at 4 °C and 11,600× *g*. HPLC measurements were performed as described in Section 2.6.

To calculate the absolute bioavailability of the oral formulation Equation (2) [32], an intravenous (IV) pharmacokinetic profile was established. Therefore, CorA was prepared as a liquid formulation comprising propylene glycol (20%), Kolliphor^®^ HS-15 (20%) and PBS pH 7.4 (60%). This CorA-solution was then administered intravenously (36 mg/kg CorA, 5 mL/kg) in the tail vein. The parameters were calculated using the pharmacokinetic software GastroPlus^®^ Version 9.7 and PKPlus^™^ Version 2.5 (SimulationsPlus Inc., Lancaster, CA, USA, 2019) applying a non-compartmental as well as a two-compartmental approach.

(2) $$F_{\mathrm{abs}}\left(\%\right)=100\times \frac{{\mathrm{AUC}}_{\left(0-\inf\right)}\;\mathrm{PO}}{{\mathrm{AUC}}_{\left(0-\inf\right)}\;\mathrm{IV}}$$

Equation (2). F_abs_ = absolute bioavailability (%), AUC_(0-inf)_ IV = area under the curve after intravenous administration, AUC_(0-inf)_ PO = area under the curve after per oral administration.

## 3. Results

### 3.1. Content Assay of CorA by ^1^H NMR

**Figure 3:**
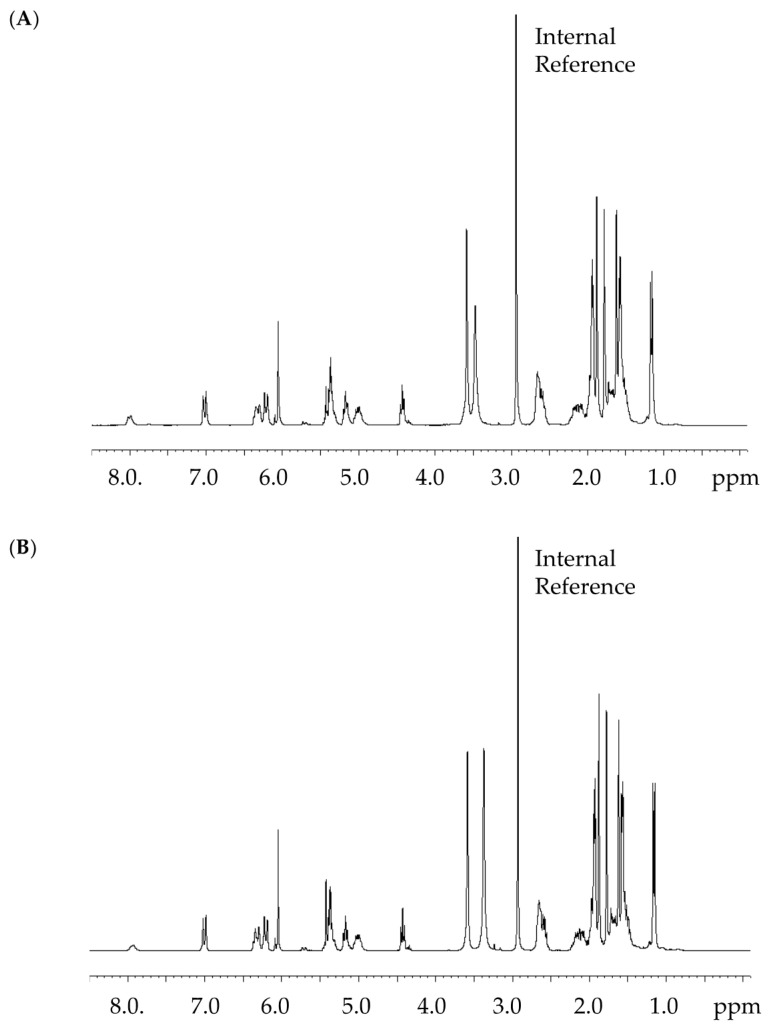
^1^H NMR spectra (300 MHz) of CorA batch A (**A**) and B (**B**) in acetonitrile-*d*_3_/D_2_O (6:1) with the internal reference compound dimethyl sulfone.

The absolute content of CorA batch (A) produced by *Corallococcus coralloides* B035 was measured by ^1^H NMR and analyzed to be 99% (Figure 3A): This batch was used in the pharmacokinetic study in mice. Batch (B) produced by the heterologous host *Myxococcus xanthus* DK1622 pDPO mxn16 Tpase [25] was analyzed to be 93% (Figure 3B): This batch was used for the in vitro tests.

### 3.2. Physicochemical Characterization of CorA via Pions’s T3 Apparatus

CorA acted as an acid in aqueous solution and had a pKa value of 3.70 determined via UV-metric titration. In accordance with its specific ionization properties, CorA showed a high lipophilicity (log *P* = 5) and poor solubility (0.1 µg/mL) at low pH values (pH 1.0–3.0). The ionization taking place at higher pH values (pH ≥ 4) decreased the lipophilicity and increased the solubility of the drug, as reflected by the log *D* and solubility values (Table 2).

### 3.3. Stability Analysis by HPLC-DAD

**Figure 4:**
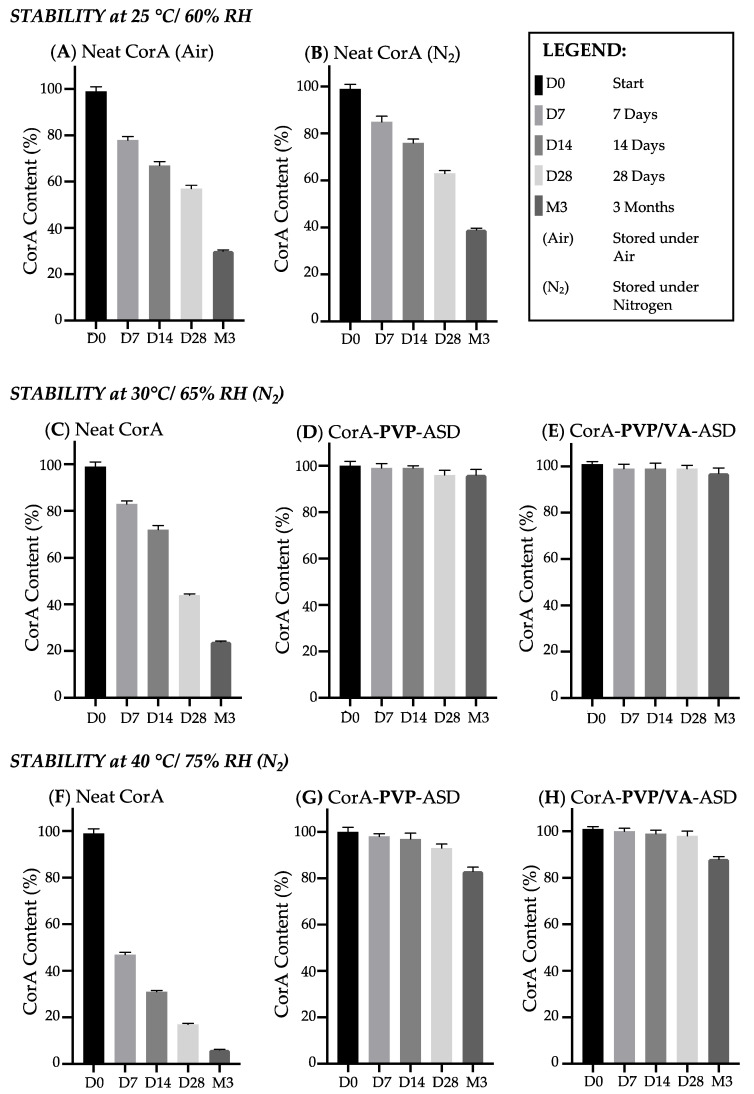
Stability analysis of neat CorA (**A**–**C**,**F**), CorA-PVP-ASD (**D**,**G**), and CorA-PVP/VA-ASD (**E**,**H**) formulations by HPLC-DAD. Samples were stored in closed twist-off glass vials with desiccant at 25 °C/60% RH (**A**,**B**), 30 °C/ 65% RH (**C**–**E**) and 40 °C/75 % RH (**F**–**H**) (*n* = 3, mean, SD).

**Figure A1:**
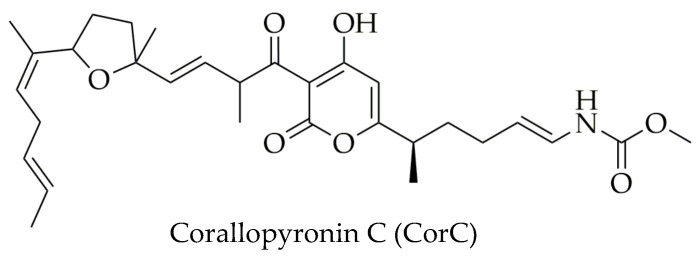
Chemical structure of corallopyronin C (CorC).

The CorA content of the neat CorA and the CorA-ASDs were analyzed directly after preparation and after being stored as described in Section 2.6. Triplicates were prepared and analyzed. The results are presented in Figure 4. Figure 4A–C,F illustrate the results of the measured neat CorA stability samples, demonstrating poor stability of the neat API. Additionally, a negative influence of air/oxygen on the stability of neat CorA was observed (Figure 4A vs. Figure 4B). When neat CorA was stored at 25 °C/60% RH under air, only 30% (±2%) of CorA remained after three months, in comparison to 39% (±2%) when stored under nitrogen. The presence of air/oxygen during the storage of the final dosage form will likely be reduced due to the compact format (tablets, capsule) compared to the neat API and the intermediate ASD powder. Further techniques like packaging under the exclusion of air/oxygen can also be applied on the final dosage form if needed. Therefore, the effect of air/oxygen was minimized for further stability analyses by storing the samples under nitrogen. The results of the neat CorA samples stored under nitrogen at different temperatures showed a correlation between the stability of CorA and the storage temperature. In comparison to the 39% (±2%) CorA measured at 25 °C/60% RH after three months, 24% (±1%) were measured at 30 °C/65% RH and only 6% (±3%) were measured at 40 °C/75% RH. CorA degradation followed a first order kinetic. The HPLC-DAD chromatograms of all neat CorA stability samples showed an increase of the isomer CorC (Figure A1).

The results of the spray-dried CorA-PVP-ASD stability analysis (Figure 4D,G) demonstrated a great increase in stability compared to the neat CorA. When CorA was embedded in PVP, 96% (±2%) were measured compared to 24% (±1%) of the neat CorA stored at 30 °C/65% RH after three months. For the CorA-PVP-ASD sample at 40 °C/75% RH, a remaining content of 88% (±1%) was measured compared to 6% (±3%) for the neat CorA samples. The results of the spray-dried CorA-PVP/VA-ASD stability analysis also demonstrated a great increase in stability (Figure 4E,H) compared to the neat CorA. The results at 30 °C/65% RH were comparable to the results for the CorA-PVP-ASD, after three months 97% (±3%) CorA was measured compared to 96% (±2%) for the CorA-PVP-ASD. Similarly to the neat CorA, CorA-ASD formulations followed first order degradation kinetics. At 40 °C/75% RH for the CorA-PVP/VA 83% (±2%) was measured compared to 88% (±1%) for the CorA-PVP-ASD. In contrast to the neat CorA, no increase of the isomer CorC was detected for both CorA-ASD formulations.

### 3.4. DSC Analysis of Neat CorA and CorA-ASD Formulations

**Figure 5:**
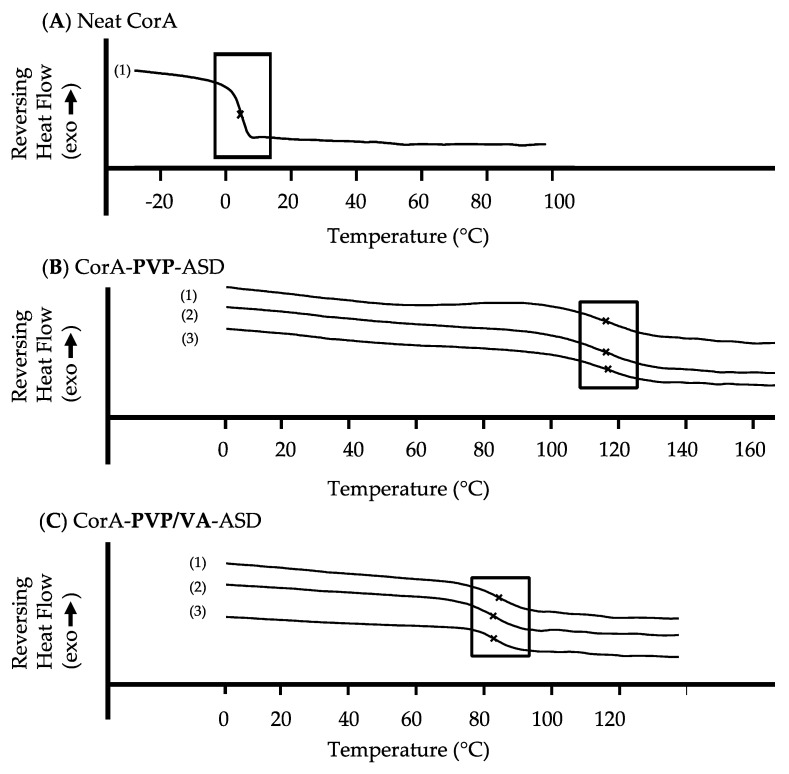
Glass transition temperature (*T*g) of the neat CorA (**A**) and CorA-PVP-ASD (**B**), CorA-PVP/VA-ASD (**C**) formulations (1) and stability analysis testing after three months at 25 °C/60% RH (2) and 40 °C/ 75% RH (3).

The glass transition temperature (*T*g) of the neat API CorA, the neat polymers PVP/VA and PVP and the CorA-ASD formulations comprising PVP and PVP/VA were measured. For stability analyses the CorA-ASD formulations were stored at 25 °C/60% RH and 40 °C/75% RH. All DSC measurements, demonstrated only one *T*g for each sample (Figure 5). No increase, decrease or additional *T*gs or melting points were found for the samples during the test period of three months. The increase of the *T*g of the CorA-ASD formulations in comparison to the neat CorA is shown in Table 3.

### 3.5. FT-IR Spectral Comparison of the Neat Polymers and CorA-ASD Formulations

**Figure 6:**
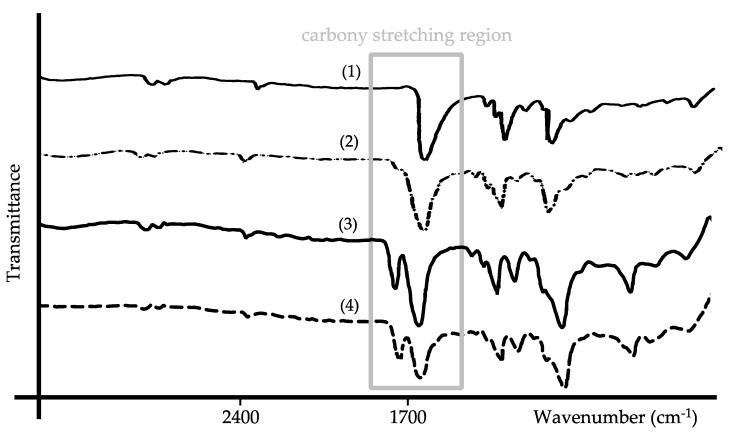
Fourier-transform-infrared (FT-IR) spectra of PVP (1), the CorA-PVP-ASD formulation (2), PVP/VA (3), and the CorA-PVP/VA-ASD formulation (4).

FT-IR spectra were measured for the neat polymer PVP and the spray-dried CorA-PVP-ASD formulation (Figure 6A). The frequencies of the bands of the neat PVP and the CorA-PVP-ASD formulation were comparable, differences were detected regarding the shape of the carbonyl stretching region at 1652 cm^−1^ (amide) [33], indicating weak molecular interactions between CorA and PVP.

For the neat polymer PVP/VA and the spray-dried CorA-PVP/VA-ASD formulations the bands were comparable regarding their frequencies (Figure 6B), differences were detected regarding the shape of the carbonyl stretching regions at 1731 cm^−1^ (ester) and 1668 cm^−1^ (amide) [34], indicating weak molecular interactions between CorA and PVP/VA.

### 3.6. Biphasic Dissolution Results of Neat CorA and CorA-ASD Formulations via BiPHa+

**Figure 7:**
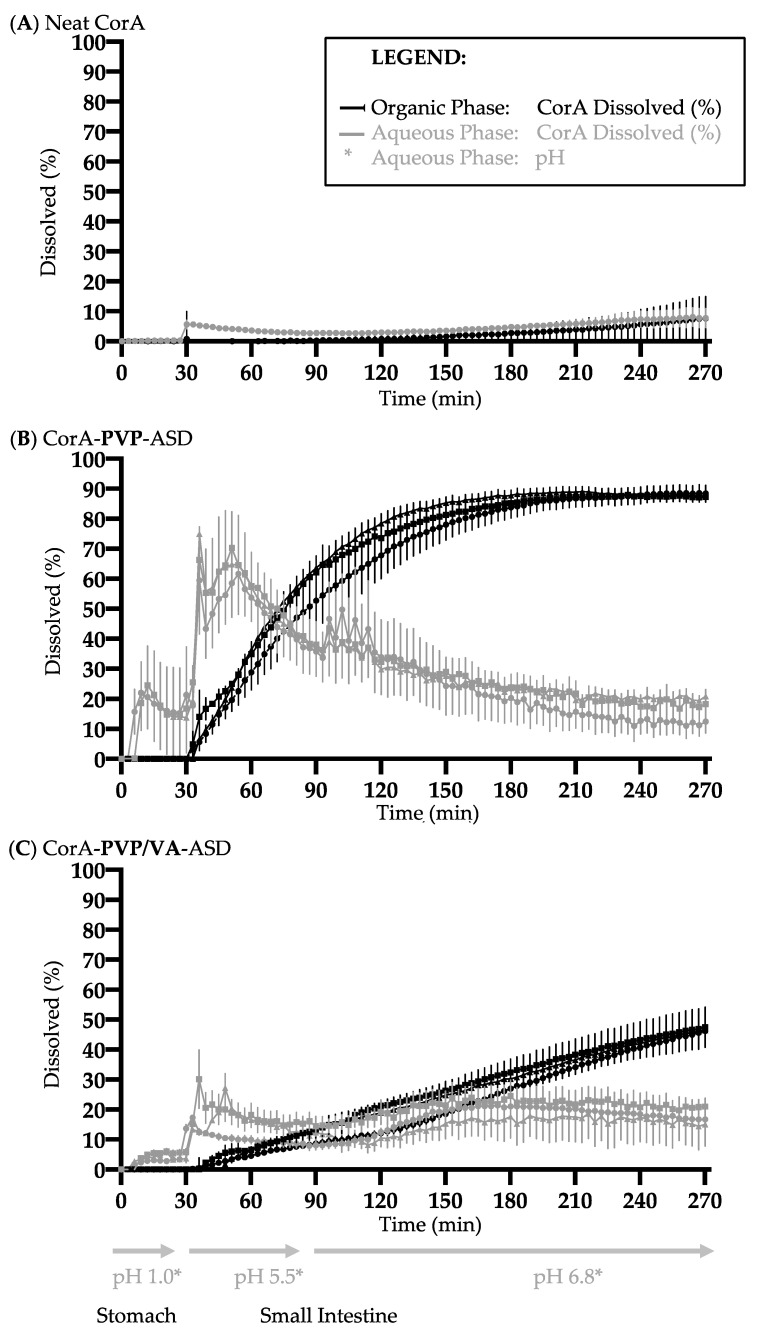
Biphasic dissolution profiles of neat CorA (**A**), spray-dried CorA-PVP-ASD formulation (**B**) and spray-dried CorA-PVP/VA-ASD formulation (**C**). Start sample (symbol = circle), four-week stability sample 25 °C/60% RH (symbol = square), four-week stability sample 40 °C/75% RH (symbol = triangle).

Biphasic dissolution tests were performed with neat CorA (Figure 7A), spray-dried CorA-PVP-ASD formulation (Figure 7B), and spray-dried CorA-PVP/VA-ASD formulation (Figure 7C). Biphasic dissolution tests of the two CorA-ASD formulations were repeated after four weeks storage at 25 °C/60% RH and 40 °C/75% RH, to determine the physicochemical stability of the CorA-ASD formulations.

Less than 1% (<2 µg/mL) of the neat CorA sample was dissolved in the aqueous phase during the gastric simulation (pH 1.0, 30 min) (Figure 7A). During the simulated small intestine passage (pH 5.5, 60 min and pH 6.8, 180 min) less than 10% (<20 µg/mL) CorA partitioned into the organic phase.

The CorA concentration of the CorA-PVP-ASD formulation in the aqueous phase during the gastric simulation (pH 1.0, 30 min) was approx. 15% (30 µg/mL) (Figure 7B). During the simulated small intestine passage (pH 5.5, 60 min and pH 6.8, 180 min) approx. 90% (180 µg/mL) CorA partitioned into the organic phase. The repetition of the biphasic dissolutions of the CorA-ASD formulations after four weeks at 25 °C/60% RH and 40 °C/75% RH provided comparable results. The CorA concentration of the CorA-PVP/VA-ASD formulation in the aqueous phase during the gastric simulation (pH 1.0, 30 min) reached approx. 5% (10 µg/mL) (Figure 7C). During the simulated small intestine passage (pH 5.5, 60 min and pH 6.8, 180 min) approx. 50% (100 µg/mL) partitioned into the organic phase. The repetition of the biphasic dissolutions of the CorA ASD formulations after four weeks at 25 °C/60% RH and 40 °C/75% RH provided comparable results.

### 3.7. Pharmacokinetic Study Results in BALB/c Mice

A pharmacokinetic study was performed in BALB/c mice (*n* = 4). Table 4 shows the pharmacokinetic parameters after the IV administration of a liquid CorA formulation and the PO administration of the solid CorA-PVP-ASD formulation. The median area under the concentration-time over all time (AUC_0-inf_) was calculated for the IV application to be 127.7 (110.2–149.0) µg·h/mL (median, IQR) and for the PO application as 75.9 (70.4–76.9) µg·h/mL (median, IQR). Based on the AUC_0-inf_ results the absolute bioavailability after the PO was calculated to be 59 (55.1–60.2) % (median, IQR) [32].

**Figure 8:**
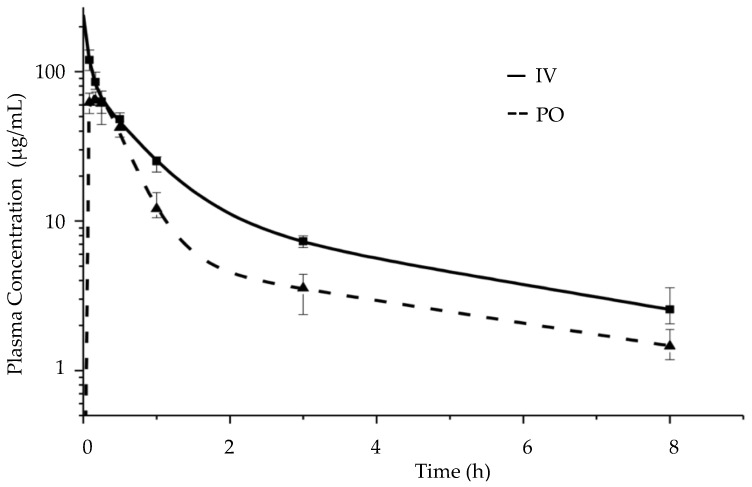
Plasma concentration time profile after IV (liquid CorA formulation) and PO (spray-dried CorA-PVP-ASD formulation) administration in BALB/c mice (median, IQR).

The maximum concentration (C_max_) after the IV administration was 119.6 (103.7–136.7) µg/mL (median, IQR) at the first time point at 5 min, the concentrations measured at the following time points decreased in value as expected after IV administration as a function of drug elimination. The measured C_max_ after PO administration was 64.3 (61.8–70.7) µg/mL (median, IQR) at the second time point at 10 min. Based on the Akaike information criterion and Schwarz criterion (both implemented in the pharmacokinetic software GastroPlus^®^ Version 9.7 and PKPlus^™^ Version 2.5) the two-compartment model was found to be optimal to describe the plasma concentration time profiles after IV and PO administration of CorA [35,36,37]. For that, each compartment displayed a first order elimination kinetic (two apparent slopes in the semi logarithmic scale (Figure 8)).

## 4. Discussion

### 4.1. Increasing the Stability and Solubility of CorA: Choice of a Suitable Solid Formulation Principle and Technique for the API

The measured poor aqueous solubility (Table 2 and Figure 7) and the estimated suitable permeability (Table 2) results indicated that the decisive factor for oral bioavailability of CorA was not permeability but solubility. Therefore, the ASD formulation principle was chosen as a promising formulation strategy based on its reported ability of dissolution, solubility and stability enhancement demonstrated for other different poorly soluble and/or instable APIs [17,18,19,20,21,22,23]. The selected water-soluble polymers PVP and PVP/VA (Figure 1B,C) are well investigated and characterized polymers for ASD formulations. Due to the semisolid, adhesive, waxy consistency of CorA, a formulation process which includes an initial dissolving step was found to be optimal to generate homogenous final solid products. Spray-drying was, therefore, chosen as suitable formulation technology. Due to the API’s poor solubility in water and good solubility in organic solvents, ethanol was selected as solvent regarding the spray-drying process. Another advantage of the spray-drying technique is the reported suitability for thermosensitive APIs [38] like CorA for which a correlation between temperature and instability was detected (Figure 4A–C,F). The resulting spray-dried powder intermediate enables further development, including easier handling for further preclinical trials.

### 4.2. Stability Analysis and Mechanism of the CorA-ASD Formulations: Via HPLC-DAD, DSC, FT-IR, and Biphasic Dissolution

The stability of the formulation is crucial for using the CorA formulation in upcoming preclinical and clinical trials and as a market product. Besides regulatory requirements regarding the stability, among other things, the efficacy of the API can be impaired when degradation products or isomers are formed. For stability testing, the neat CorA and the spray-dried ASD formulations comprising PVP and PVP/VA were stored at different temperatures and the content was analyzed by HPLC-DAD (Figure 4). The instability of CorA in the presence of oxygen (Figure 4A vs. Figure 4B) is most likely due to the auto-oxidation of the double bond system. The process of auto-oxidation can cause the formation of hydro-peroxides, which later decompose to aldehydes and ketones.

**Figure 9:**
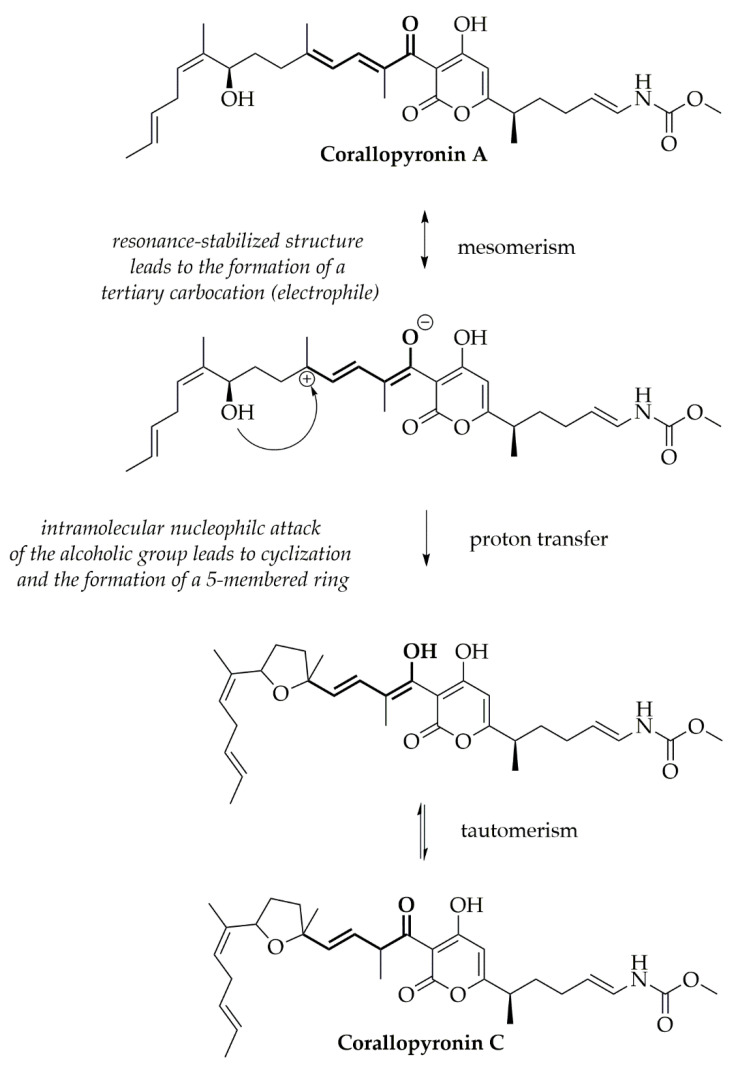
Postulated isomerization of CorA to CorC due to an intramolecular nucleophilic attack of the alcoholic group.

The analysis of the neat CorA stored in the presence of air or nitrogen resulted in the detection of an increased isomerization of CorA into CorC when stored at temperatures ≥ 25 °C. The mechanism of the isomerization from CorA towards CorC was, therefore, analyzed and the tertiary allylic carbocation was found to be the likely driving force, for a nucleophilic attack of the alcoholic group, resulting in cyclization and generation of CorC (Figure 9).

**Figure 10:**
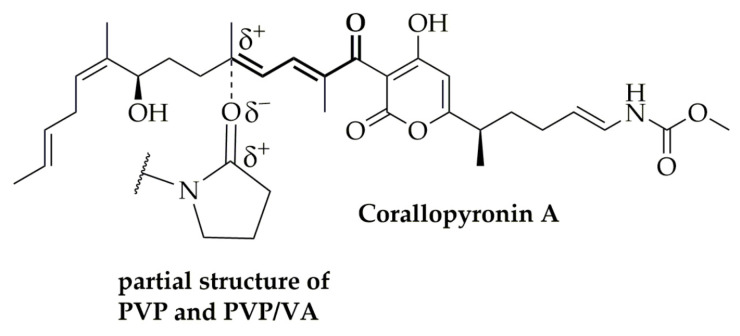
Postulated molecular interactions of CorA with the polymers PVP and PVP/VA.

The isomerization towards CorC was suppressed when CorA was embedded in the polymers PVP or PVP/VA. One likely mechanism stabilizing CorA (Figure 10), in which the amide structure of PVP and PVP/PV and the ester structure of PVP/VA interact with CorA via Debye forces that stabilize the carbocation and suppress isomerization towards CorC. The vibrational FT-IR spectra of the neat PVP in comparison to the CorA-PVP-ASD formulation and neat PVP/VA in comparison to the CorA-PVP/VA formulation show characteristic bands for the polymers PVP and PV/VA and demonstrate no changes regarding the frequencies of the carbonyl stretching regions (Figure 6). Changes in frequencies of the carbonyl stretching regions have been found to be indicators for hydrogen bonding between polymers like PVP and APIs. The absence of the phenomenon indicates the potential presence of other molecular interactions, like the postulated Debye forces [39] (Figure 10).

Additionally, a likely second stabilization mechanism of CorA, when embedded in PVP or PVP/VA, is due to the reduced molecular mobility and increased glass transition temperature (*T*g) of the spray-dried CorA-ASD formulations compared to the neat CorA (Table 3, Figure 5). Amorphous APIs or amorphous formulations are often reactive and instable to mechanical and thermal stresses above their *T*g. Hancock et al. [21] found it was necessary to cool to at least 50 K below the experimental *T*g before the molecular motions detected by DSC could be considered to be negligible over the lifetime of a typical pharmaceutical product. The *T*g of the neat CorA was determined to be 5 °C. At storage conditions of 25 °C, based on the findings of Hancock et al. [21], CorA is highly reactive and instable which corresponds with the stability results of CorA (Figure 4A,B). The increase in *T*g from 5 °C of the neat CorA to 116 °C of the CorA-PVP-ASD formulation and 84 °C for the CorA PVP/VA ASD formulation resulted in stable products at 25 and 30 °C for the test period of three months, which corresponds to the rule of thumb presented by Hancock et al. [21].

Moreover, the DSC curves of the CorA-PVP and CorA-PVP/VA-ASD formulation, including the stability samples, only showed one *T*g (Figure 5), indicating no phase separation, a potential destabilization driving force of ASD formulations [40].

Furthermore, the absence of melting points indicated that the sample was of pure amorphous character. Since the amorphous state is often instable due to its higher energy level, crystallization tendencies need to be investigated. Subsequently, formulations need to be chosen which are able to suppress crystallization during the respected product cycle, since aqueous solubility and therefore bioavailability can be comprised by API crystallization [41]. For both CorA-ASD formulations comprising PVP and PVP/VA no crystallization was detected by DSC for the test period of three months at 25 °C/60% RH and 40 °C/75% RH (Figure 5). However, as CorA never revealed any tendency to crystalize, physical instability would have shown rather an amorphous/amorphous phase separation. Biphasic dissolutions were performed directly after sample preparation and when stored for one month at 25 °C/60% RH and 40 °C/75% RH. The biphasic dissolution results indicated no physicochemical changes of the formulation (Figure 7), which would have affected the dissolution or solubility properties during the test.

### 4.3. Dissolution and Solubility Analysis and Mechanism of the CorA-ASD Formulations: Biphasic Dissolution Apparatus BiPHa+ and Pion’s T3 Apparatus

**Figure 11:**
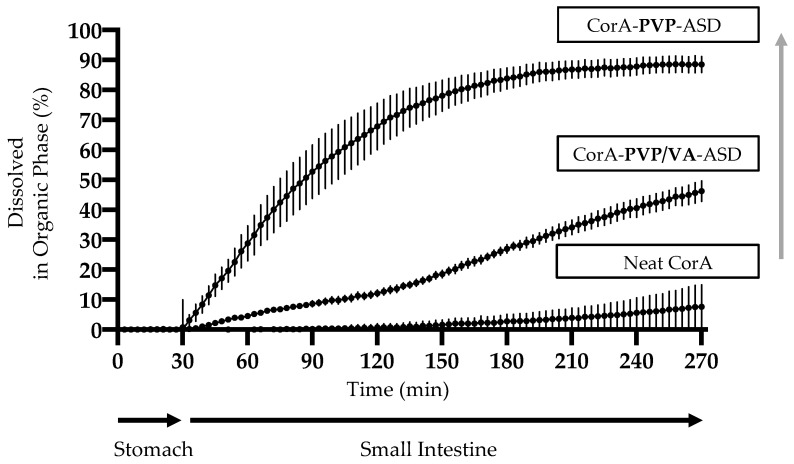
Increase of fraction absorbed into organic phase: Neat CorA < CorA-PVP/VA ASD formulation < CorA-PVP ASD formulation.

Biphasic dissolution was selected as an in vitro tool predictive for the in vivo situation, measuring the fraction absorbed in the organic phase, as an estimate and surrogate parameter for the fraction absorbed into the human gut wall, and subsequently the human bloodstream, after oral administration [24,42,43,44]. The fraction dissolved during the stomach passage at pH 1.0 showed an increase in solubility due to the ASD formulation principle. The fraction of neat CorA dissolved stayed below 1% (<2 µg/mL) during the 30 min stirred at 37 °C, and increased to 15% (30 µg/mL) when CorA-was embedded into PVP, and 5% (10 µg/mL) when CorA was embedded in PVP/VA. The total CorA fraction absorbed during the simulated small intestinal passage of neat CorA was below 10% (<20 µg/mL) and increased to approx. 90% (180 µg/mL) when CorA was embedded in PVP, and to approx. 50% (100 µg/mL) when CorA was embedded in PVP/VA (Figure 11). The course of the biphasic dissolution curve of the CorA-PVP intermediate powder demonstrated a fast dissolution in the aqueous FASSIF-like medium at pH 5.5 and 6.8 (Figure 7B and Figure 10), which correlated with a fast partitioning into the organic phase. The course of the biphasic dissolution of the spray-dried CorA-PVP/VA-ASD formulation (Figure 7C and Figure 10) demonstrated the pH-dependent solubility of CorA which was also measured during solubility measurements of the neat API (Table 2). The slope of the linear dissolution curve of the CorA PVP/VA-ASD in the organic phase was lower at pH 5.5 compared to pH 6.8 and the dissolution curve also demonstrated a slower dissolution rate, compared to the CorA-PVP-ASD, and did not reach its plateau within the test duration of 4.5 h (Figure 7C and Figure 11).

The increase in surface area by spray-drying and the good wettability properties of the selected polymers are probable drivers for the detected increase in dissolution and solubility of CorA, phenomena already described for other poorly soluble APIs. During the spray-drying process, the adhesive CorA substance was converted into a solid powder form. The wettability constitutes an essential initial step before any dissolution. Therefore, two polymers (PVP and PVP/VA) with good wetting characteristics where selected, with PVP being superior to PVP/VA in terms of wettability [45,46]. Verma and Rudraraju [46] found a correlation of wetting kinetics and the dissolution rates of PVP and PVP/VA-ASD formulations, which probably accounts for the superior performance of the PVP-ASD. The better wettability of PVP resulted in a faster disintegration of the CorA-PVP-ASD particles and led to a faster dissolution. A second difference between PVP and PVP/VA is the enhanced binding ability of the former. Furthermore, different gelation kinetics of the polymers may cause distinct dissolution profiles of the CorA-PVP-ASD and CorA-PVP/VA-ASD. For the latter, a gelation process including the formation of clusters of enclosed dry CorA-PVP/VA-ASD formulation by a gel-layer was observed, which may be responsible for reduced dissolution rate of the ASD formulation. Prior mixing of the CorA-PVP/VA-ASD with lactose as diluting excipient to reduce the formation of clusters, resulted in dissolution profiles comparable to the CorA-PVP-ASD formulation. These results demonstrated the possibility to apply the CorA-PVP/VA-ASD for a retard release in humans. An immediate release can either be achieved by the addition of diluting excipients to the CorA-PVP/VA-ASD or by the use of PVP as polymer.

The increased biphasic dissolution and solubility profiles of the CorA formulations compared to neat CorA strongly indicated the improved oral bioavailability of the API, given sufficient permeability of the API. As a guide for good intestinal permeability Lipinski suggests a filter of log *D* > 0 and < 3. For CorA log *D* values < 3 were measured for pH values above 6.0, which indicated good intestinal permeability at higher pH values (Table 2).

### 4.4. Pharmacokinetic Analysis of the CorA-ASD Formulation: Investigations in BALB/c Mice

The in vivo pharmacokinetic study of the PO administration of the spray-dried CorA-ASD formulation comprising PVP was performed in BALB/c mice. The fast course and high values (C_max_, T_max_ and AUC) of the PO curve demonstrated suitable CorA solubility and permeability achieved by the CorA-PVP-ASD formulation principle (Figure 8). Good oral bioavailability (59%) was determined, which is a crucial factor regarding the intended solid oral dosage form.

Whether the high C_max_ and AUC values achieved with the CorA-PVP-ASD formulation correlate with good in vivo efficacy in human or whether better results can be achieved by the CorA-PVP/VA-ASD formulation depends on the concentration of the drug in relation to the minimum inhibitory concentration for the pathogen, e.g., *Wolbachia*, and on the time this exposure is maintained [47]. Future preclinical and clinical trials will answer this question. The chosen formulation principle enables altering the dissolution as needed, e.g., by increasing the particle size of the CorA-PVP-ASD formulation by granulation or switching to a rather zero order kinetics using the CorA-PVP/VA-ASD. Thus, tailor-made release profiles would be available.

## 5. Conclusions

The highly anti-infective, but semisolid and adhesive, poorly water-soluble, and instable active pharmaceutical ingredient CorA was formulated into amorphous solid dispersions comprising the water-soluble polymers PVP or PVP/VA. The spray-dried ASD formulations led to solid powders with increased stability, dissolution, and solubility characteristics in vitro. The in vivo administration of the spray-dried CorA-PVP-ASD formulation to BALB/c mice supported the in vitro results, indicating a suitable oral bioavailability of the prepared CorA formulations. The ASD formulation via spray drying was found to be a suitable oral formulation strategy for preclinical, clinical, and market products for the anti-infective CorA.

## 6. Patents

The University of Bonn is the patent applicant for the following patents for the use of corallopyronin A for filarial infections: US 9168244 B2, US 9687470 B2, EP 2704708 B1. The authors A.H., K.P., A.S., S.K. and G.M.K. are the inventors of these patents.

## Figures and Tables

**Table 1:** CorA-amorphous solid dispersion (ASD) formulation excipients and process parameters.

Excipients and Parameters	CorA-PVP-ASD	CorA-PVP/VA-ASD
CorA	1.6022 g	1.6008 g
Polymer	6.4237 g	6.4032 g
Organic Solvent (Ethanol 99%)	52.930 g	52.931 g
Theoretical Active Ingredient Content	20%	20%
Inlet Temperature	85 °C	85 °C
Outlet Temperature	59 °C	59 °C
Flow Rate	5.6 mL/min	4.5 mL/min

**Table 2:** PH-dependent solubility, partition coefficient (log *P*) and distribution coefficient (log *D*) of neat CorA determined by Pion’s T3 apparatus.

pH	Solubility (µg/mL)	Log *D*
1.0	0000.11	5.42 → log *P* (neutral XH)
2.0	0000.11	5.41
3.0	0000.14	5.31
4.0	0000.40	4.85
5.0	0002.98	3.97
6.0	0028.86	3.00
6.5	0091.13	2.52
7.0	0288.00	2.09
7.4	0723.20	1.81
8.0	2874.00	1.54

**Table 3:** Glass transition temperature (*T*g) of the neat CorA, CorA-PVP-ASD and CorA-PVP/VA-ASD formulations, and the neat polymers PVP and PVP/VA.

API and Excipients	*T*g (°C)
Neat CorA	5
CorA-PVP-ASD formulation (20% drug load)	116
Neat PVP	160
CorA-PVP/VA-ASD formulation (20% drug load)	84
Neat PVP/VA	110

**Table 4:** Results of the pharmacokinetic analysis after IV administration of a CorA-solution and PO administration of the solid CorA-PVP-ASD formulation in BALB/c mice (*n* = 4).

Pharmacokinetic Parameters	IV Median (IQR)	PO Median (IQR)
AUC_(0–8h)_ (µg·h/mL)	115.5 (102.4–127.1)	67.8 (60.8–71.6)
AUC_(0-inf)_ (µg·h/mL)	127.7 (110.2–149.0)	75.9 (70.4–76.9)
C_max_ (µg/mL)	119.6 (103.7–136.7)	64.3 (61.8–70.7)
T_max_ (min)	5 *	10 (10–11.3)
F_abs_ (%)	100 **	59 (55.1–60.2)

* first measured value; ** IV result median set to 100%.

## Appendix A

**Table A1:** Gradient CorA HPLC-DAD method.

Time (min)	A (%)	B (%)	Time (min)	A (%)	B (%)
0	70	30	17.00	40	60
1.50	70	30	19.50	40	60
5.00	60	40	23.00	30	70
7.50	60	40	25.50	30	70
11.00	50	50	29.00	20	80
13.50	50	50	30.00	20	80
